# Dihydroartemisinin attenuates lipopolysaccharide-induced osteoclastogenesis and bone loss via the mitochondria-dependent apoptosis pathway

**DOI:** 10.1038/cddis.2016.69

**Published:** 2016-03-31

**Authors:** C Dou, N Ding, J Xing, C Zhao, F Kang, T Hou, H Quan, Y Chen, Q Dai, F Luo, J Xu, S Dong

**Affiliations:** 1 Department of Orthopedics, Southwest Hospital, Third Military Medical University, Chongqing 400038, China; 2 Department of Biomedical Materials Science, School of Biomedical Engineering, Third Military Medical University, Chongqing 400038, China; 3 China Orthopedic Regenerative Medicine Group, Chongqing 400038, China

## Abstract

Dihydroartemisinin (DHA) is a widely used antimalarial drug isolated from the plant *Artemisia annua*. Recent studies suggested that DHA has antitumor effects utilizing its reactive oxygen species (ROS) yielding mechanism. Here, we reported that DHA is inhibitory on lipopolysaccharide (LPS)-induced osteoclast (OC) differentiation, fusion and bone-resorption activity *in vitro*. Intracellular ROS detection revealed that DHA could remarkably increase ROS accumulation during LPS-induced osteoclastogenesis. Moreover, cell apoptosis was also increased by DHA treatment. We found that DHA-activated caspase-3 increased Bax/Bcl-2 ratio during LPS-induced osteoclastogenesis. Meanwhile, the translocation of apoptotic inducing factor (AIF) and the release of cytochrome *c* from the mitochondria into the cytosol were observed, indicating that ROS-mediated mitochondrial dysfunction is crucial in DHA-induced apoptosis during LPS-induced osteoclastogenesis. *In vivo* study showed that DHA treatment decreased OC number, prevents bone loss, rescues bone microarchitecture and restores bone strength in LPS-induced bone-loss mouse model. Together, our findings indicate that DHA is protective against LPS-induced bone loss through apoptosis induction of osteoclasts via ROS accumulation and the mitochondria-dependent apoptosis pathway. Therefore, DHA may be considered as a new therapeutic candidate for treating inflammatory bone loss.

Bone is a dynamic organ that undergoes continuous remodeling throughout life. Bone homeostasis is maintained by a balanced bone-resorbing and bone-forming process. In this process, hematopoietic stem cells or monocytes/macrophage progenitor cell-derived osteoclasts (OCs) are mainly responsible for bone resorption.^1^ Abnormal OC function is associated with numerous diseases, and most of them are due to excessive osteoclastic activity. These diseases include osteoporosis, rheumatoid arthritis and periodontitis.^2, 3^ Two of the most important regulating factors during OC differentiation are receptor activator of nuclear factor *κ*B ligand (RANKL) and macrophage-colony-stimulating factor (M-CSF).^4, 5^ Binding of RANKL to RANK results in the initiation of the TNF receptor-associated factor 6 signaling, which activates nuclear factor-*κ*B, Akt and MAP kinase (ERk, JNK and p-38), and eventually leads to the proliferation, differentiation and maturation of OCs.^6, 7^

Lipopolysaccharide (LPS) is an important component of the outer membrane of Gram-negative bacteria. In LPS-induced bone loss, many factors are involved including local host response, prostanoids and cytokine production, inflammatory cell recruitment and OC activation.^8, 9, 10^ Experimental evidence have shown that LPS-mediated inflammation is highly dependent on reactive oxygen species (ROS) and the associated downstream MAPK signaling pathways including ERK, JNK and p-38.^11, 12^ ROS has been shown having an important role in the process of OC differentiation, survival, activation and bone resorption.^13, 14, 15, 16^ It has also been proved that ROS production in OC and intracellular hydrogen peroxide accumulation is critical for osteoclastogenesis and skeletal homeostasis.^17^ Recently, a study reported that LPS induces OC formation via the ROS-mediated JNK and STAT3 pathway, which could be blocked by peroxiredoxin II.^18^

Dihydroartemisinin (DHA) is the main active metabolite isolated from the plant *Artemisia annua*. DHA has been widely used as first-line therapeutics against falciparum malaria.^19^ Recent evidence suggested that DHA has antitumor effects because of its unique cytotoxicity mechanism.^20^ In particular, studies reported that DHA is pro-apoptotic in tumor cell lines regarding breast and prostate cancer.^21, 22^ Although the detailed mechanism of DHA cytotoxicity and pro-apoptotic effects is not fully understood, DHA-mediated ROS production has a central role.^23, 24^ However, the effect of DHA on bone health has not been studied.

In the present study, we reported that DHA could attenuate LPS-induced OC differentiation, fusion and bone-resorption activity *in vitro*. Our data showed that DHA-induced cell apoptosis during LPS-induced osteoclastogenesis via intracellular ROS generation and mitochondria-mediated pathways. DHA administration in LPS-induced mouse models decreased OC number and reversed bone loss *in vivo*.

## Results

### DHA inhibits LPS-induced OC differentiation

**Figure 1:**
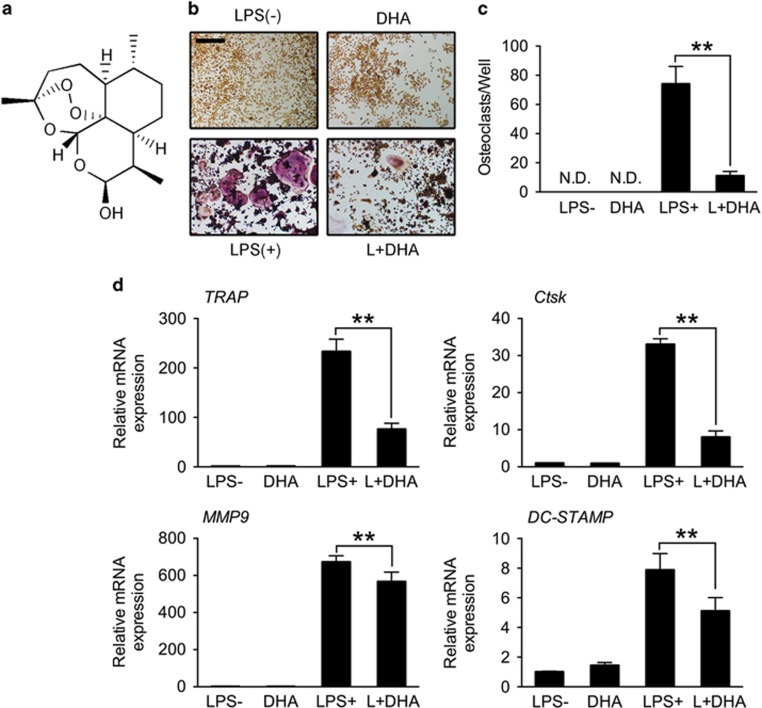
DHA inhibits LPS-induced osteoclast differentiation. (**a**) Chemical formula of DHA. (**b**) Representative images of preosteoclasts stained for TRAP (red) treated with LPS (1*μ*g/ml), DHA (100 *μ*g/ml) or both for 3 days. Scale bar represents 200 *μ*m. (**c**) Quantification of TRAP (+) cells with more than three nuclei in each well (96-well plate). The data in the figures represent the averages±S.D. (**d**) Relative mRNA expression levels of *TRAP*, *Ctsk, MMP-9* and *DC-STAMP* of BMMs in different groups on day 3. The data in the figures represent the averages±S.D. Significant differences between the treatment and control groups are indicated as *P<0.05 or **P<0.01

We first evaluated the toxicity of LPS and found that LPS can decrease cell viability (100 ng/ml), increase cell apoptosis rate (1000 ng/ml) and induce cell cycle arrest (10 ng/ml) in bone marrow macrophages (BMMs; Supplementary Figure S1). However, LPS at the dose of 1000 ng/ml could stimulate the highest OC number in BMMs (Supplementary Figure S4). On the basis of this, we use LPS of 1000 ng/ml in the following studies. The chemical formula of DHA is shown (Figure 1a). The cytotoxicity of DHA was evaluated by CCK-8 assay and cell apoptosis assay, suggesting that DHA dose at 100 *μ*g/ml had no cytotoxicity in BMMs (Supplementary Fig. S5). Tartrate-resistant acid phosphatase (TRAP) stain was then performed to test the effects of DHA on LPS-induced osteoclastogenesis (Figure 1b). TRAP-positive (red) cells with more than three nuclei were counted as osteoclasts. Quantification analysis revealed that DHA significantly decreased LPS-induced OC number (*P*<0.01; Figure 1c). In accordance, qPCR results also showed that mRNA expression of OC-specific marker including *TRAP*, *Ctsk*, *MMP-9* and *DC-STMAP* were significantly suppressed by DHA treatment (*P* <0.01; Figure 1d).

### DHA inhibits LPS-induced OC fusion and actin ring formation

**Figure 2:**
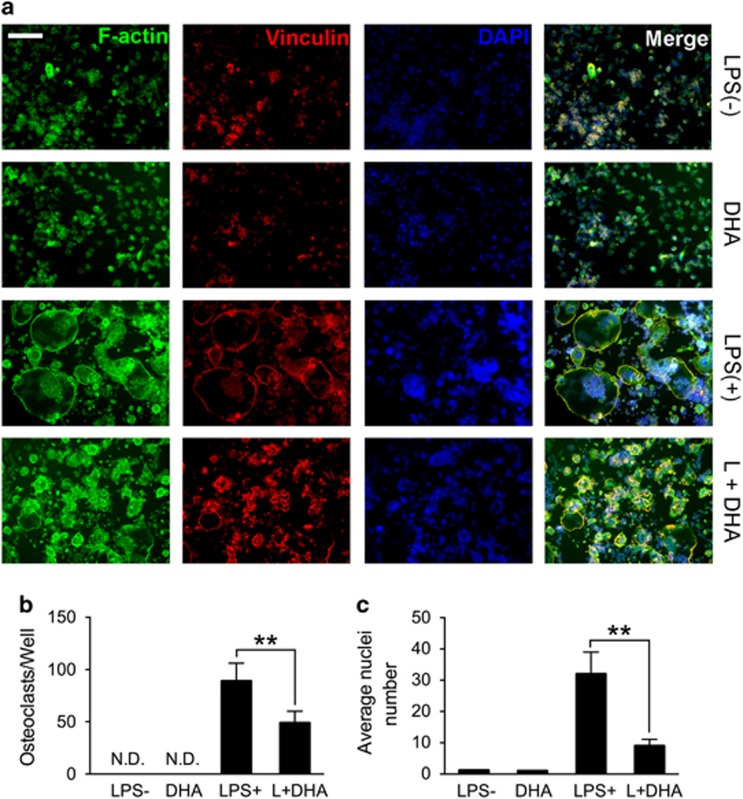
DHA inhibits LPS-induced osteoclast fusion and actin ring formation. (**a**) Representative images of focal and adhesion staining of preosteoclasts treated with LPS (1 *μ*g/ml), DHA (100 *μ*g/ml) or both for 3 days. Scale bar represents 100 *μ*m. (**b**) Quantification of osteoclasts (nuclei⩾3) in each well (96-well plate). The data in the figures represent the averages±SD. (**c**) Quantification of average nucleus number in each group. The data in the figures represent the averages±S.D. Significant differences between the treatment and control groups are indicated as *P<0.05 or **P<0.01

To further study the effects of DHA on LPS-induced OC formation, actin cytoskeleton and focal adhesion stain was performed for better visualization of the actin ring. From the stain results we figured out that actin ring formation was hindered by DHA treatments (Figure 2a). Consistent with TRAP stain results, OC number was decreased by DHA treatment (*P*<0.01; Figure 2b). In addition, average nucleus number of OCs was also significantly decreased by DHA treatments (*P*<0.01), suggesting that OC fusion was also inhibited (Figure 2c).

### DHA inhibits LPS-induced OC bone-resorption activity

**Figure 3:**
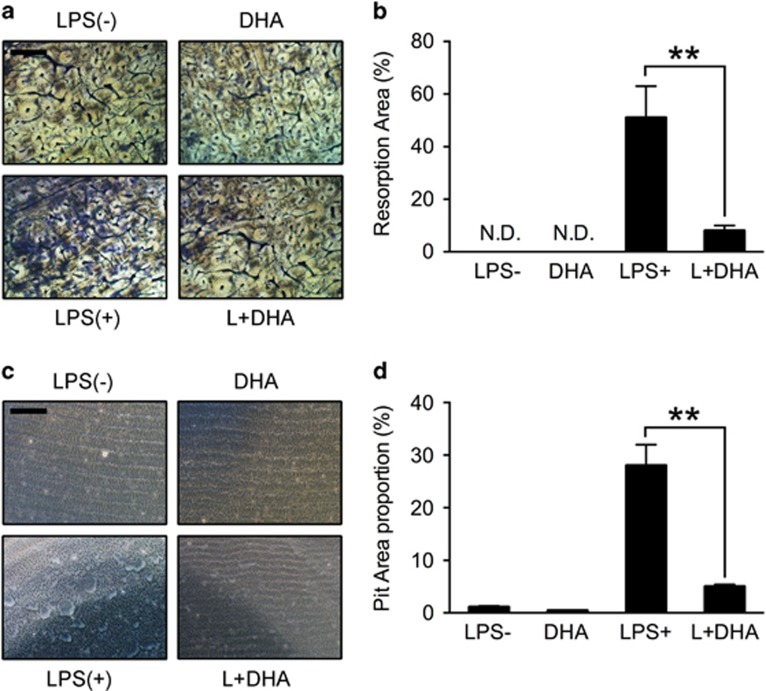
DHA inhibits LPS-induced osteoclast bone-resorption activity. (**a**) Representative images of preosteoclasts cultured on bovine bone slices treated with LPS (1 *μ*g/ml), DHA (100 *μ*g/ml) or both for 5 days. Scale bar represents 400 *μ*m. (**b**) Quantification of bone-resorption area on the bone slices. The data in the figures represent the averages±S.D. (**c**) Representative images of Osteoassay surface 96-well plate after removal of osteoclasts. Scale bar represents 400 *μ*m. (**d**) Quantification of bone-resorption area on the osteo surface. The data in the figures represent the averages±S.D. Significant differences between the treatment and control groups are indicated as *P<0.05 or **P<0.01

To further test the effects of DHA on LPS-induced OC function, pit formation assay was performed on bone slices and osteo surface (Figures 3a and c). BMMs were seeded on bone slices and osteo surface according to group settings. After 5 days' incubation, cells were removed and resorption pits were quantified. Consistent with previous results, DHA showed strong inhibitory effects on OC resorption activity on both bone slices and osteo surface (*P* <0.01; Figures 3b and d).

### DHA increases intracellular ROS generation and cell apoptosis rate during LPS-induced osteoclastogenesis

**Figure 4:**
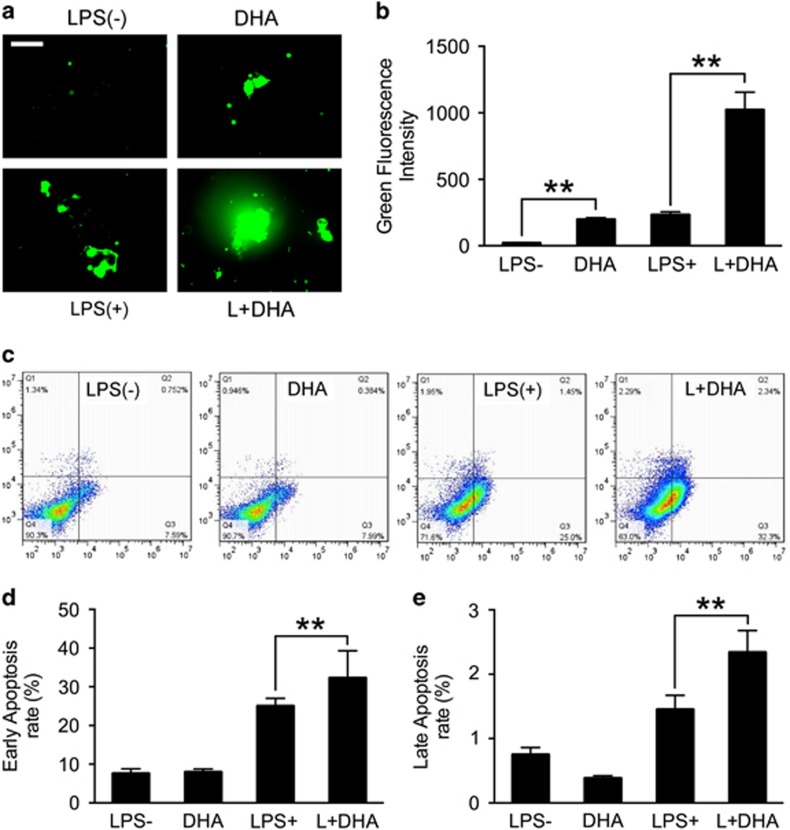
DHA increases intracellular ROS generation and cell apoptosis rate during LPS-induced osteoclastogenesis. (**a**) Representative images of ROS-positive preosteoclasts treated with LPS (1 *μ*g/ml), DHA (100 *μ*g/ml) or both for 3 days. Scale bar represents 100 *μ*m. (**b**) Quantification of ROS green fluorescence intensity in each well (96-well plate). The data in the figures represent the averages±S.D. (**c**) FCM analysis of the cell apoptosis rate of preosteoclasts treated with LPS (1 *μ*g/ml), DHA (100 *μ*g/ml) or both for 3 days. (**d**) Quantification analysis of the early-stage cell apoptosis rate. The data in the figures represent the averages±S.D. (**e**) Quantification analysis of the late-stage cell apoptosis rate. The data in the figures represent the averages±S.D. Significant differences between the treatment and control groups are indicated as *P<0.05 or **P<0.01

To explain the inhibitory effects of DHA on osteoclastogenesis, intracellular ROS was detected using 2′, 7′-dichlorofluorescein diacetate (DCFH)-DA (Figure 4a). BMMs treated with DHA alone showed higher ROS intensity compared with the control group (*P*<0.01). Cells treated with LPS alone also exhibited upregulated ROS intensity; however, BMMs treated with both LPS and DHA showed significantly higher ROS intensity compared with other groups (Figure 4b). Annexin-V/PI staining was then used to test the effects of DHA on cell apoptosis (Figure 4c). Quantification analysis showed that LPS treatment alone showed the higher early and late cell apoptosis rate compared with control groups (Figures 4d and e). The apoptosis rate induced by LPS was further increased with DHA treatments (*P*<0.01; Figures 4d and e). The results suggested that DHA could increase intracellular ROS intensity and apoptosis rate in the presence of LPS.

### DHA induces apoptosis in LPS-induced osteoclastogenesis through the mitochondria-dependent pathway

**Figure 5:**
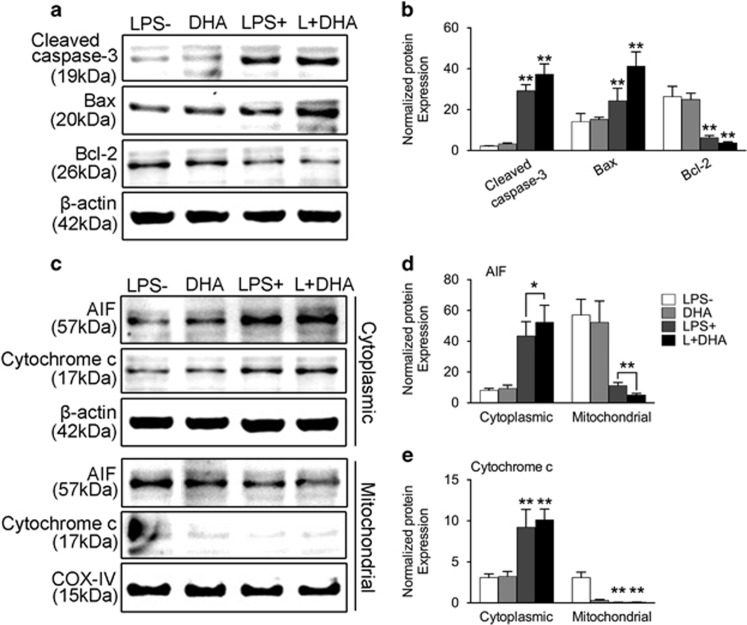
DHA induces apoptosis in LPS-induced osteoclasts through the mitochondria-dependent pathway. (**a**) Representative western blot images of cleaved caspase-3, Bax, Bcl-2 and *β*-actin from preosteoclasts treated with LPS (1 *μ*g/ml), DHA (100 *μ*g/ml) or both for 3 days. (**b**) Quantification of normalized protein expression intensity of caspase-3, Bax and Bcl-2. The data in the figures represent the averages±S.D. (**c**) Representative western blot images of AIF, cytochrome *c* and *β*-actin in the cytosol; AIF, Cytochrome *c* and COX-IV in mitochondria. (**d**) Quantification of normalized protein expression intensity of AIF in the cytosol and mitochondria. The data in the figures represent the averages±S.D. (**e**) Quantification of normalized protein expression intensity of cytochrome *c* in the cytosol and mitochondria. The data in the figures represent the averages±S.D. Significant differences between the treatment and control groups are indicated as *P<0.05 or **P< 0.01

To investigate the underlying mechanisms of DHA-induced apoptosis in LPS-induced osteoclastogenesis, western blot analysis was performed to detect the expression of apoptosis-related proteins in preosteoclasts of different groups after 24-h culture (Figure 5a). As shown in Figure 5b, the protein expression of Bax and activated caspase-3 were remarkably increased, whereas the expression of Bcl-2 was significantly decreased in groups treated with both LPS and DHA. As the accumulation of ROS is crucial for mitochondrial apoptosis,^25^ we further detected pro-apoptotic proteins including cytochrome *c* and apoptotic inducing factor (AIF) released from mitochondria to the cytosol (Figure 5c). Notably, when preosteoclasts were treated with LPS and DHA together, the expression level of AIF and cytochrome *c* decreased in the mitochondria and increased in the cytosol (Figures 5 and d). The results indicated that DHA-induced apoptosis during LPS-induced osteoclastogenesis was associated with mitochondrial dysfunction. It is well recognized that cytochrome *c* activates apoptosis effector caspase-3, which is consistent with results showed in Figure 5a.^26^

### DHA reduces LPS-induced osteoclastogenesis and bone loss *in vivo*

**Figure 6:**
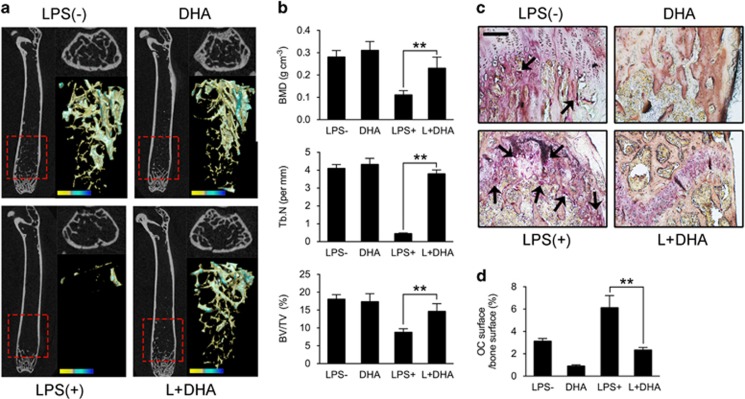
DHA reduces LPS-induced osteoclastogenesis and bone loss *in vivo.* (**a**) Representative *μ*CT longitudinal section images of the femurs, cross-sectional view of the distal femurs and reconstructed trabecular structure of the ROI (red dashed box). Color scale bar represents the bone mineral density level. (**b**) Quantitative *μ*CT analysis of distal femoral volumetric bone mineral density, BV/TV and trabecular number (Tb. N) in each group. The data in the figures represent the averages±S.D. (**c**) Representative images of histological slides of TRAP stain focusing on the metaphyseal region of the distal femur from mice of different groups. Scale bar represents 800 *μ*m. (**d**) Quantitative analysis of OC surface/bone surface ratio. The data in the figures represent the averages±S.D. Significant differences between the treatment and control groups are indicated as *P<0.05 or **P<0.01

To investigate whether the *in vitro* effects of DHA still function *in vivo*, we used LPS-induced bone-loss mouse model. Mice were injected with LPS intraperitoneally and treated with DHA or vehicle. Mice were killed after 4 weeks of treatment. *μ*CT analysis was performed using dissected femurs. For trabecular bone analysis of the distal femur, an upper 3-mm region beginning 0.8 mm proximal to the most proximal central epiphysis of the femur was contoured showed with red dashed box (Figure 6a). Quantification analysis showed that DHA administration in LPS-induced bone-loss mouse models could significantly increase bone mineral density, trabecular bone volume fraction (BV/TV) and trabecular number (Tb. N; *P*<0.01; Figure 6b). TRAP stain of the distal femur showed that OC surface/bone surface ratio was significantly higher in LPS-treated mice compared with the control group (Figure 6c). DHA administration remarkably decreased the OC surface ratio (Figure 6d). The *in vivo* results indicated that DHA administration could attenuate LPS-induced osteoclastogenesis and bone loss.

## Discussion

DHA is famous for its efficient therapeutic effects in treating malaria. In recent years, accumulating evidence showed that DHA inhibits cell growth, arrests cell cycle at the G0/G1 phase and induces apoptotic cell death in different kinds of tumor cells.^27, 28, 29^ It has been proved that the cytotoxicity of DHA is based on ROS and carbon-centered radical generation.^30^ The role of ROS during RANKL-induced osteoclastogenesis is essential. MAPK including JNK and p-38 are crucial for OC differentiation, and both of these two genes can be activated by ROS.^15, 31^ Numerous reports were made on the inhibitory effects in osteoclastogenesis of antioxidant compounds.^32, 33^ Current evidence suggest that most antioxidants could inhibit osteoclastogenesis via scavenging ROS. Although the reports are few, excessive intracellular ROS generation in OCs could also cause oxidative damage and eventually lead to DNA damage and cell apoptosis.^34^ In this regard, we were curious about the effects of DHA in osteoclastogenesis.

Many kinds of inflammatory bone loss are due to LPS.^10^ LPS produces a significant amount of ROS during the inflammatory process, which contributes to accelerated OC differentiation and activation.^35^ The *in vitro* research of our study showed that LPS could significantly increase the intracellular ROS level during osteoclastogenesis (Figure 4a). When DHA was introduced into the LPS-mediated osteoclastogenesis process, the ROS level was further elevated (Figure 4a). Accordingly, the apoptosis rate increased along with the ROS accumulation (Figures 4d and e). ROS have many sources, including the mitochondrial electron transport chain, xanthine oxidase, cytochrome P-450 enzymes, uncoupled NO synthases and NADPH oxidases.^18, 36^ As mitochondria are susceptible to oxidative damage, which lead to enhanced mitochondrial ROS generation,^37^ we further examined the mitochondrial apoptotic factors including cytochrome *c* and AIF from both inner mitochondria and the cytosol. Cytochrome *c* in the cytosol activates caspase-8 and caspase-9, which further activate executioner caspase-3 to induce cell apoptosis.^38^ In our study, DHA treatment together with LPS significantly increased cytochrome *c* and AIF release from mitochondria to the cytosol and activated caspase-3. In addition, pro-apoptotic protein Bax expression was increased and anti-apoptotic protein Bcl-2 was decreased, leading to an increased ratio of Bax/Bcl-2 (Figures 5a and b). Taken together, these results strongly indicated that DHA induces OC apoptosis in LPS-induced osteoclastogenesis through accumulation of ROS and the mitochondrial apoptotic pathway.

It is interesting to notice that DHA alone could not induce cell apoptosis in BMMs (Supplementary Figure S6); it was only pro-apoptotic in the presence of LPS. To explain this, one should notice that the redox balance in the culture condition regulates *in vitro* LPS-induced OC formation.^39^ An antioxidant enhances LPS-induced OC formation, whereas a pro-oxidant reduces it. DHA is known for its induction of iron-dependent oxidative stress and has been proved potential to serve as a redox chemotherapeutic that selectively induces cell apoptosis.^40^ As ROS generation is necessary during OC differentiation, combined with our results, we concluded that OCs are sensitive to DHA-induced apoptosis because of upregulation of cellular oxidative stress. In contrast, BMMs have low cellular oxidative stress before their differentiation toward OCs, which make them less sensitive to DHA-induced apoptosis. Very recently, two studies reported that DHA could also inhibit RANKL-induced osteoclastogenesis through inhibiting RANKL-induced signaling pathways.^41, 42^ It is also worthy to test whether the redox balance works in the inhibitory effects of DHA in RANKL-induced osteoclastogenesis.

In summary, the present study demonstrated that DHA treatment during LPS-induced osteoclastogenesis increased cell apoptotic death via accumulation of ROS, increased release of cytochrome *c* and AIF from mitochondria into the cytosol, activation of caspase-3 and increase in the Bax/Bcl-2 ratio. Moreover, *in vivo* studies suggested that DHA administration effectively prevented bone loss, rescued bone microarchitecture and restored bone strength in LPS-induced mouse models. Although more research is needed to explore the underlying mechanism, our data imply the protective role of DHA in LPS-induced bone loss, which could be useful in the development of new treatments for patients with inflammatory bone loss.

## Materials and Methods

### Reagents

Recombinant Mouse RANKL and Recombinant Mouse M-CSF were purchased from R&D Systems (Minneapolis, MN, USA). Antibodies against AIF, cytochrome *c*, cleaved caspase-3, Bax, Bcl-2 and *β*-actin were purchased from Santa Cruz Biotechnology (Santa Cruz, CA, USA). Osteo Assay Surface for Bone Resorption was purchased from Corning (Corning, NY, USA). Bovine cortical bone slices were obtained from Boineslices.com (Jelling, Denmark). Cell Counting Kit-8 was obtained from Dojindo Molecular Technologies (Dojindo, Japan). The TRAP stain kit was obtained from Sigma-Aldrich (St. Louis, MO, USA). The Actin Cytoskeleton and Focal Adhesion Staining Kit was purchased from Millipore (Darmstadt, Germany). The DCFDA cellular ROS detection assay kit was obtained from ABcam (Cambridge, UK). Alpha minimal essential Medium (*α*-MEM) and fetal bovine serum (FBS) were purchased from Gibco (Life Technologies, Carlsbad, CA, USA). Penicillin–streptomycin solution was obtained from Hyclone (Thermo Scientific, Waltham, MA, USA). DHA was purchased from Sigma-Aldrich.

### Mice

Eight-week-old female C57BL/6 mice were provided by the animal center of Third Military Medical University. All experimental procedures were approved by Third Military Medical University and were performed according to the guidelines of laboratory animal care and use. All efforts were made to reduce the number of animals tested and their suffering. Mice were divided into four groups: phosphate-buffered saline (PBS)-treated (LPS−), DHA (100 mg/kg) only-treated (DHA), LPS (5 mg/kg) only-treated (LPS+) and DHA (100 mg/kg)-treated LPS (5 mg/kg) groups (L+DHA). LPS and DHA dissolved in PBS was injected intraperitoneally three times a week for 4 weeks. Mice were weighed daily, and concentration was calculated for DHA administration. All treated mice were killed by cervical dislocation 1 day after last administration.

### *In vitro* assays for OC differentiation, fusion and function

Bone marrow cells were separated and cultured with M-CSF (50 ng/ml) for 24 h to obtain BMMs. BMMs were cultured in MEM containing 10% FBS and 1% penicillin–streptomycin solution. BMMs were then treated with M-CSF (30 ng/ml) and RNAKL (100 ng/ml) for 36 h to generate preosteoclasts.

For the TRAP stain, preosteoclasts were cultured in a 96-well plate at a density of 5 × 10^3^ cells/well with different treatments for 3 days. Cells were fixed in 4% paraformaldehyde for 20 min and then stained with TRAP staining solution according to the manufacturers' instructions. Relative TRAP activity was measured by colorimetric analysis.

For actin cytoskeleton and focal adhesion stain, preosteoclasts were cultured on glass sheet in a 12-well plate at a density of 4 × 10^4^ cells/well with different treatments for 3 days. Procedures were described in previous study.^43^ In brief, on day 4, cells were washed and fixed for permeabilization. After blocking, cells were incubated in primary antibody solution for 1 h at room temperature. After wash, cells were incubated with secondary antibody and TRITC-conjugated Phalloidin for 1 h at room temperature. Nucleus counterstaining was performed by DAPI for 5 min, followed by fluorescence microscopy observation.

For pit formation assay, preosteoclasts were incubated in 96-well plates (Corning Osteo Assay Surface) of 2 × 10^3^ cells/well and in 48-well plates covered with bovine bone slices of 1 × 10^4^ cells/well. Cells were induced with different treatments for 5 days. Methylene blue stain was performed to evaluate the resorption area on bone slices. Bleach solution was added to remove cells. Detailed analysis of the pit formation area was described previously.^44^

### Intracellular ROS detection

Intracellular ROS detection was performed using the DCFDA cellular ROS detection assay kit (ABcam). Preosteoclasts (5 × 10^3^ cells/well in 96-well plates) were treated with LPS (1 *μ*g/ml), DHA (100 *μ*g/ml) or both for 72 h. The intracellular ROS level was measured by 2′, 7′-dichlorofluorescein diacetate (DCFH), which can be oxidized into fluorescent DCF. Cells were washed in 1 × PBS and then incubated in the dark for 30 min with 10 *μ*M DCFH-DA. Images were taken using the fluorescence microscopy (Olympus, Tokyo, Japan).

### Annexin-V/PI staining

Cell apoptosis was determined with Annexin-V/PI staining. Preosteoclasts were cultured for 72 h with treatments of LPS, DHA or both. Cells were washed twice with cold PBS and then resuspended in 500 *μ*l of binding buffer (10 mM HEPES/NaOH (pH 7.4), 140 mM NaCl, 2.5 mM CaCl_2_) at a concentration of 1 × 10^6^ cells/ml. Cells were then stained with 5 *μ*l of annexin V-FITC (Life Technologies) and 10 *μ*l of 20 *μ*g/ml PI. Apoptosis was analyzed using a FAC Star flow cytometer (BD, Triangle, NC, USA).

### *μ*CT and histological analyses

For *μ*CT analysis,the Bruker MicroCT Skyscan 1272 system (Kontich, Belgium) with an isotropic voxel size of 10.0 *μ*m was used to image the whole femur. Scans were conducted in 4% paraformaldehyde and used an X-ray tube potential of 60 kV, an X-ray intensity of 166 *μ*A and an exposure time of 1700 ms. Trabecular bones were thresholded at 86–255 (8-bit gray scale bitmap). *μ*CT scans of the whole body of mice (except skull) were performed using isotropic voxel sizes of 148 *μ*m. Reconstruction was accomplished by Nrecon (Ver. 1.6.10, Kontich, Belgium). 3D images were obtained from contoured 2D images by methods based on distance transformation of the gray scale original images (CTvox, Kontich, Belgium, Ver. 3.0.0). 3D and 2D analyses were performed using software CT Analyser (Kontich, Belgium, Ver. 1.15.4.0). All images presented are representative of the respective groups.

For the bone histological analysis, femurs were dissected and fixed in 4% paraformaldehyde in PBS for 48 h. Femurs were then decalcified by daily change of 15% tetrasodium EDTA for 2 weeks. Tissues were dehydrated by passage through an ethanol series, cleared twice in xylene, embedded in paraffin and sectioned at 8 *μ*m thickness along the coronal plate from anterior to posterior. Decalcified femoral sections were stained with TRAP.

### RT-qPCR

Total RNA was isolated using Trizol reagent (Life Technologies). Single-stranded cDNA was prepared from 1 *μ*g of total RNA using reverse transcriptase with oligo-dT primer according to the manufacturer's instructions (Promega, Fitchburg, WI, USA). Two microlitres of each cDNA were subjected to PCR amplification using specific primers as follows: TRAP (F) 5′-AGACGAGGTTACGCTGTGC-3′, (R) 5′-TCGGGGACAATTCGGTAAACT-3′ Ctsk (F) 5′-GCGGCATTACCAACAT-3′, (R) 5′-CTGGAAGCACCAACGA-3′ MMP-9 (F) 5′-ACCCGAAGCGGACATT-3′, (R) 5′-GGCATCTCCCTGAACG-3′ DC-STAMP (F) 5′-TTATGTGTTTCCACGAAGCCCTA-3′, (R) 5′-ACAGAAGAGAGCAGGGCAACG-3′.

### Immunoblotting

Cells were lysed in a lysis buffer containing 10 mM Tris, pH 7.2, 150 M NaCl, 5 mM EDTA, 0.1% SDS, 1% Triton X-100 and 1% deoxycholic acid. For western blots, 30 *μ*g of protein samples were subjected to SDS-PAGE, followed by transfer onto PVDF membranes. After blocking in 5% skim milk, membranes were incubated with rabbit antibodies against CaMKK*β*, Pyk2, c-FOS, NFATc1, COX-IV and *β*-actin overnight at 4 °C, followed by 1-h incubation with secondary antibody (1:2000). COX-IV and *β*-actin were detected in parallel as a loading control for mitochondrial and cytoplasmic proteins, respectively.

### Statistical analysis

All data are representative of at least three experiments of similar results performed in triplicate, unless otherwise indicated. Data are expressed as mean±S.D. One-way ANOVA, followed by Student–Newman–Keuls *post hoc* tests, was used to determine the significance of difference between results, with *P<0.05, **P<0.01 being regarded as significant.

## Abbreviations

DHA: dihydroartemisinin
OC: osteoclast
RANKL: receptor activator of nuclear factor *κ*B ligand
M-CSF: macrophage-colony-stimulating factor
LPS: lipopolysaccharide
ROS: reactive oxygen species
AIF: apoptotic inducing factor
BMMs: bone marrow macrophages
TRAP: tartrate-resistant acid phosphatase
BV/TV: bone volume fraction
Tb. N: trabecular number
