# Supplementary material for: Dihydroartemisinin attenuates lipopolysaccharide-induced osteoclastogenesis and bone loss via the mitochondria-dependent apoptosis pathway
Source: Cell Death Dis. 2016 Mar 31;7(3):e2162–. doi: 10.1038/cddis.2016.69 (PMC4823966; doi:10.1038/cddis.2016.69)
Supplement: Supplementary Information [file cddis201669x1.docx]

**Supplementary Figures**

Supplementary Figure 1


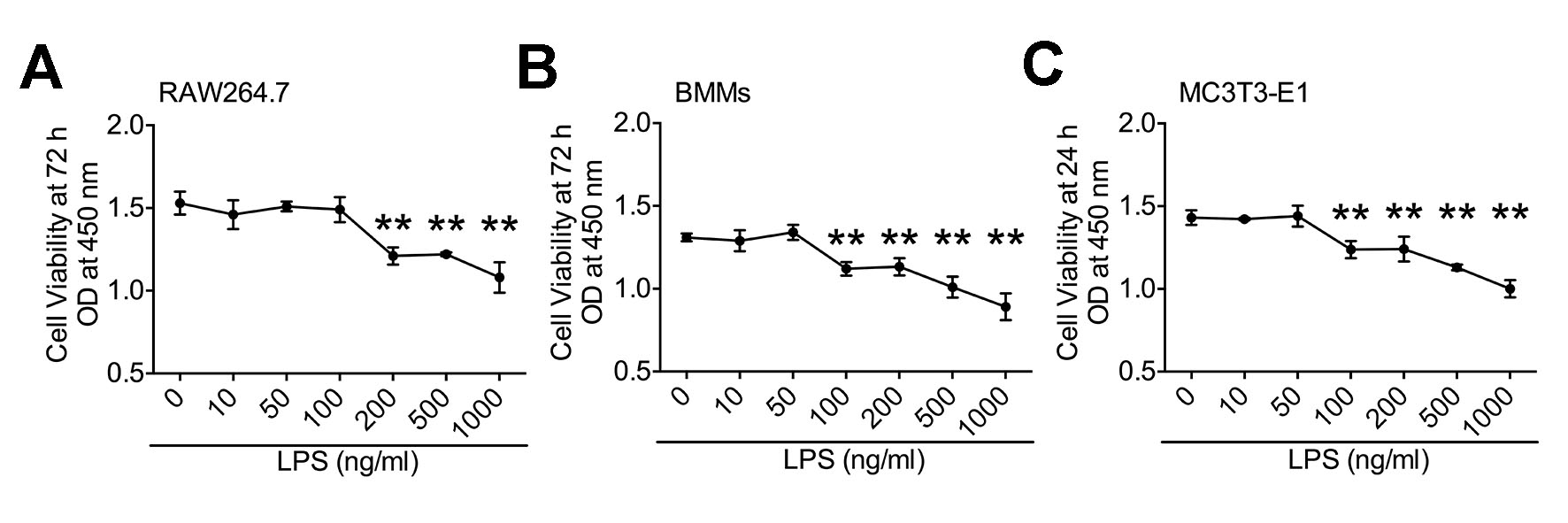


**Supplementary Fig. 1 Effects of LPS on cell viability. (A)** CCK-8 was performed in triplicate to analyze the cell viability of RAW264.7 cells treated with varying doses of LPS for 72 h. **(B)** CCK-8 was performed in triplicate to analyze the cell viability of BMMs treated with varying doses of LPS for 72 h. **(C)** CCK-8 was performed in triplicate to analyze the cell viability of MC3T3-E1 cells treated with varying doses of LPS for 72 h. The data in the figures represent the averages ± SD. Statistically significant differences between the treatment and control groups are indicated as * (*p*< 0.05) or ** (*p*< 0.01).

Supplementary Figure 2


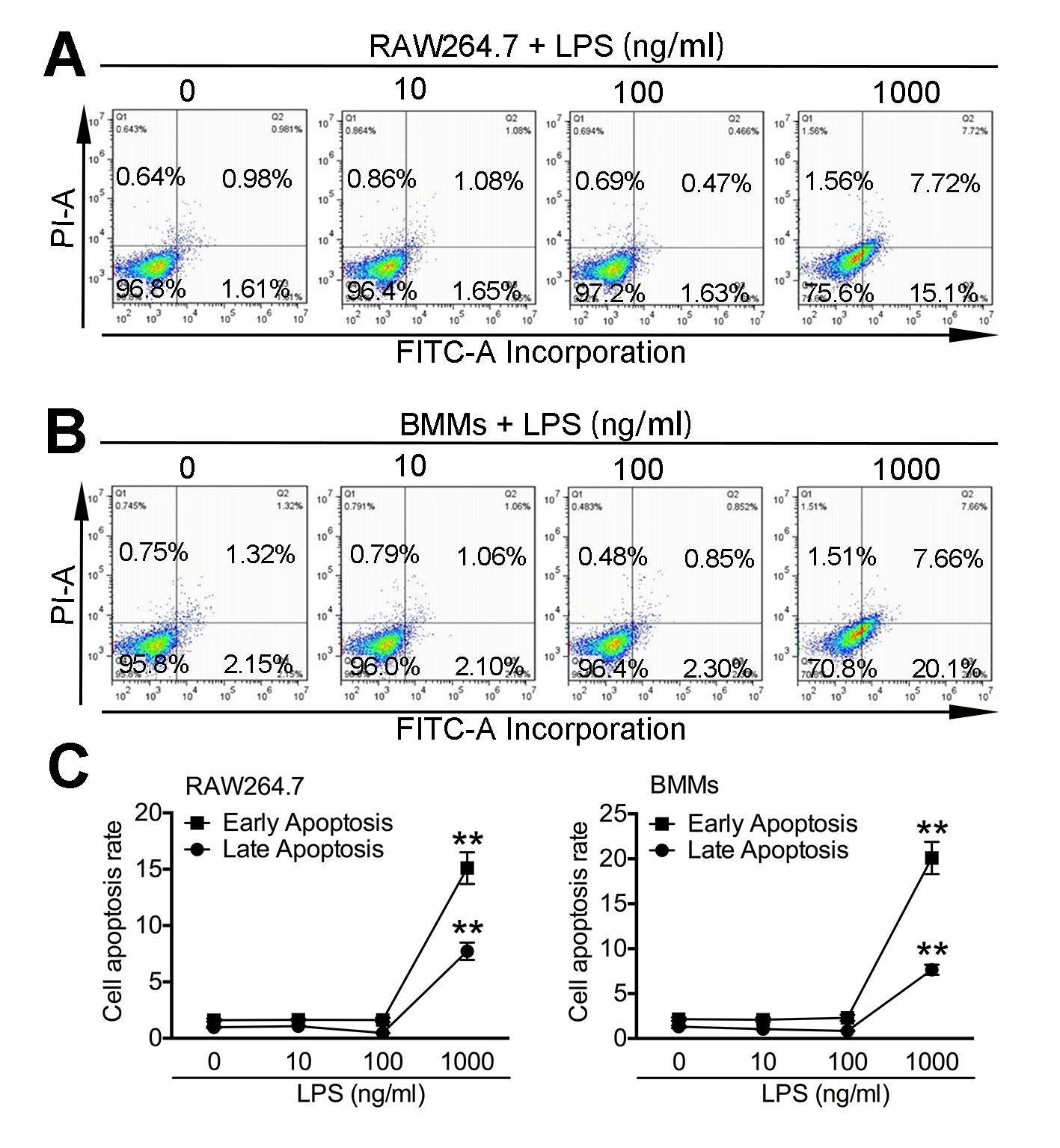


**Supplementary Fig. 2 Effects of LPS on cell apoptosis. (A)** FCM analysis of the apoptosis rate of RAW264.7 cells treated with LPS for 72 h. **(B)** FCM analysis of the apoptosis rate of BMMs treated with LPS for 72 h. **(C)** Quantitative analysis of the early and late stage apoptosis rates. The data in the figures represent the averages ± SD. Statistically significant differences between the treatment and control groups are indicated as * (*p*< 0.05) or ** (*p*< 0.01).

Supplementary Figure 3


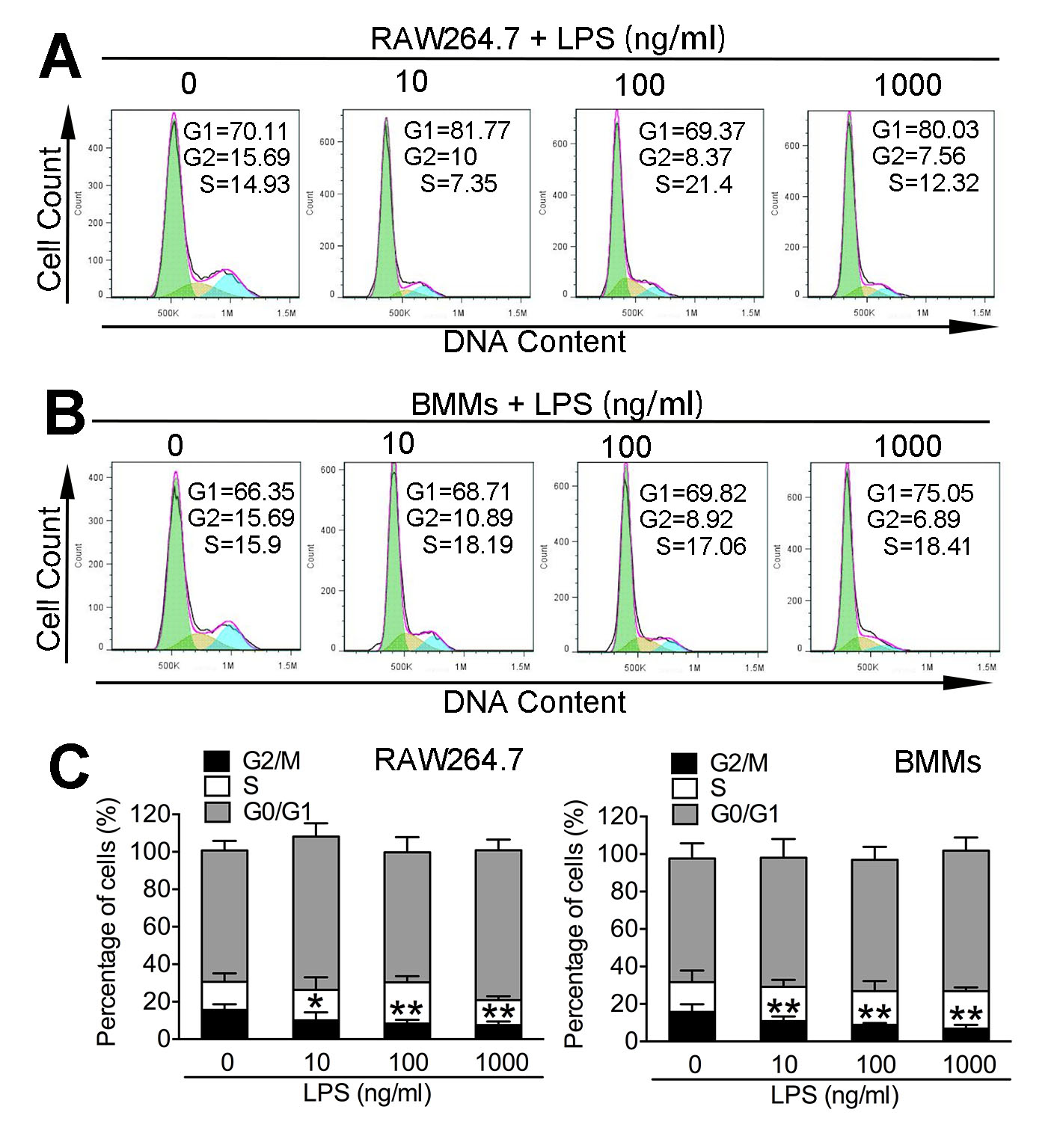


**Supplementary Fig. 3 Effects of LPS on cell apoptosis. (A)** FCM analysis of the cell cycle change of RAW264.7 cells treated with LPS for 72 h. **(B)** FCM analysis of the cell cycle change of BMMs treated with LPS for 72 h. **(C)** Histogram showing cell cycle changes of RAW264.7 cells and BMMs treated with LPS. The data in the figures represent the averages ± SD. Statistically significant differences between the treatment and control groups are indicated as * (*p*< 0.05) or ** (*p*< 0.01).

Supplementary Figure 4


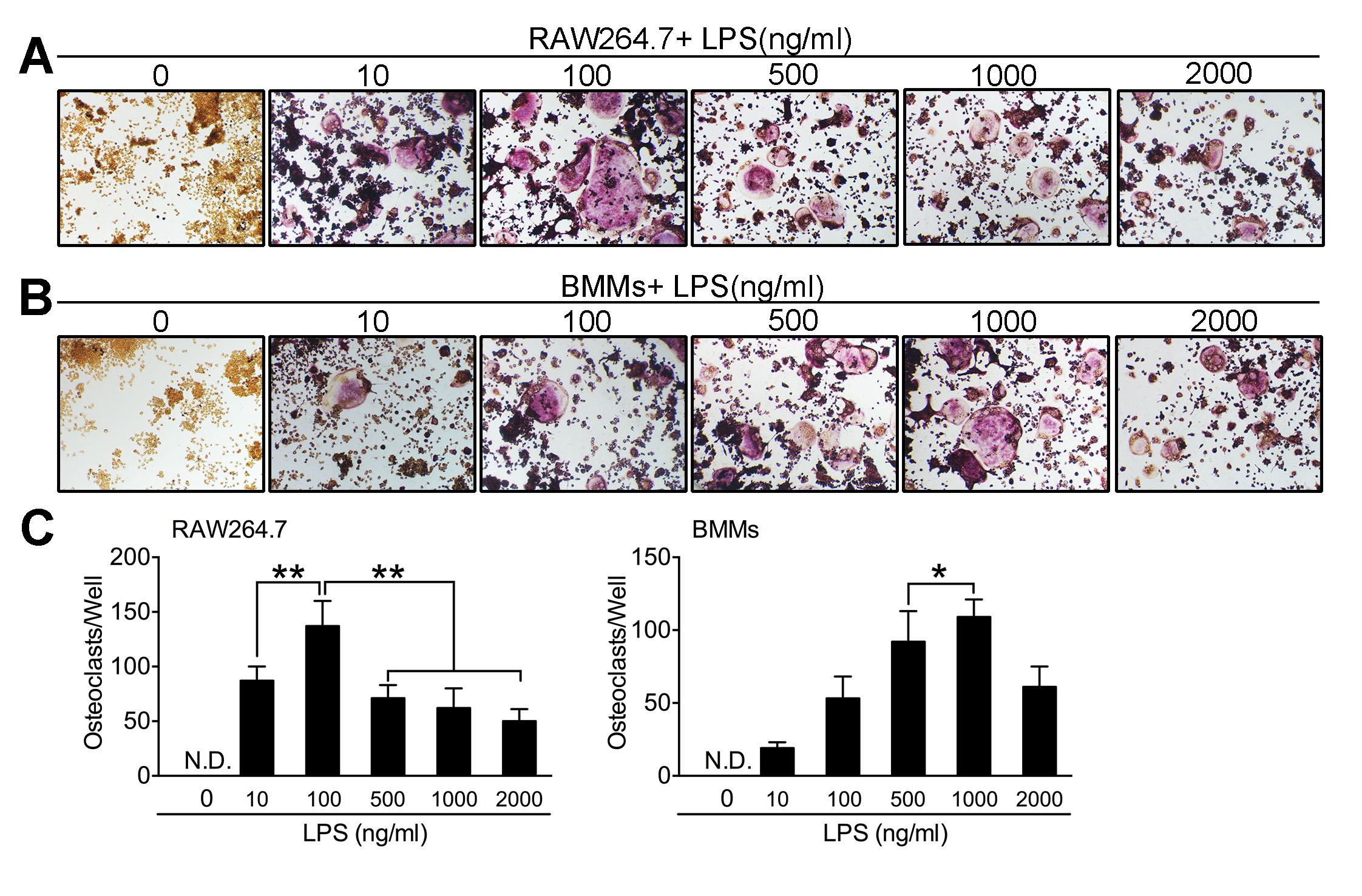


**Supplementary Fig. 4 LPS sensitivity of RAW264.7 cells and BMMs in osteoclastognesis. (A)** Representative images of RAW264.7 cells stained for TRAP (red) treated with LPS at different doses. Experiments were done in triplicate. **(B)** Representative images of BMMs stained for TRAP (red) treated with LPS at different doses. Experiments were done in triplicate. **(C)** Quantification of TRAP (+) cells with more than three nuclei (osteoclast) in each well (96-well plate). The data in the figures represent the averages ± SD. Statistically significant differences between the treatment and control groups are indicated as * (*p*< 0.05) or ** (*p*< 0.01).

Supplementary Figure 5


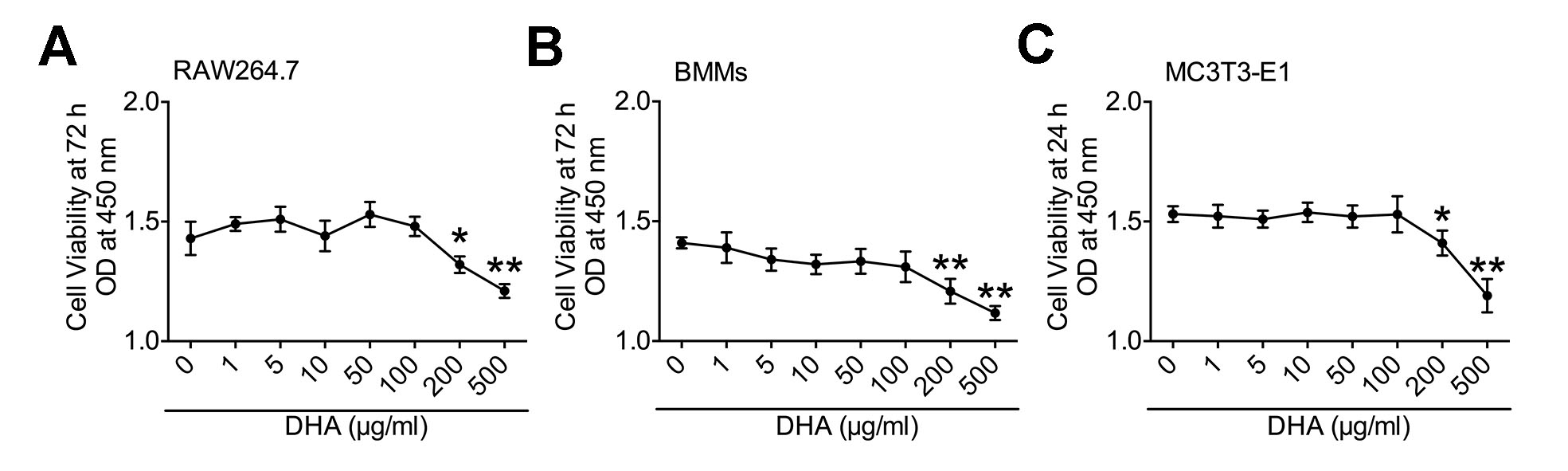


**Supplementary Fig. 5 Effects of DHA on cell viability. (A)** CCK-8 was performed in triplicate to analyze the cell viability of RAW264.7 cells treated with varying doses of DHA for 72 h. **(B)** CCK-8 was performed in triplicate to analyze the cell viability of BMMs treated with varying doses of DHA for 72 h. **(C)** CCK-8 was performed in triplicate to analyze the cell viability of MC3T3-E1 cells treated with varying doses of DHA for 72 h. The data in the figures represent the averages ± SD. Statistically significant differences between the treatment and control groups are indicated as * (*p*< 0.05) or ** (*p*< 0.01).

Supplementary Figure 6


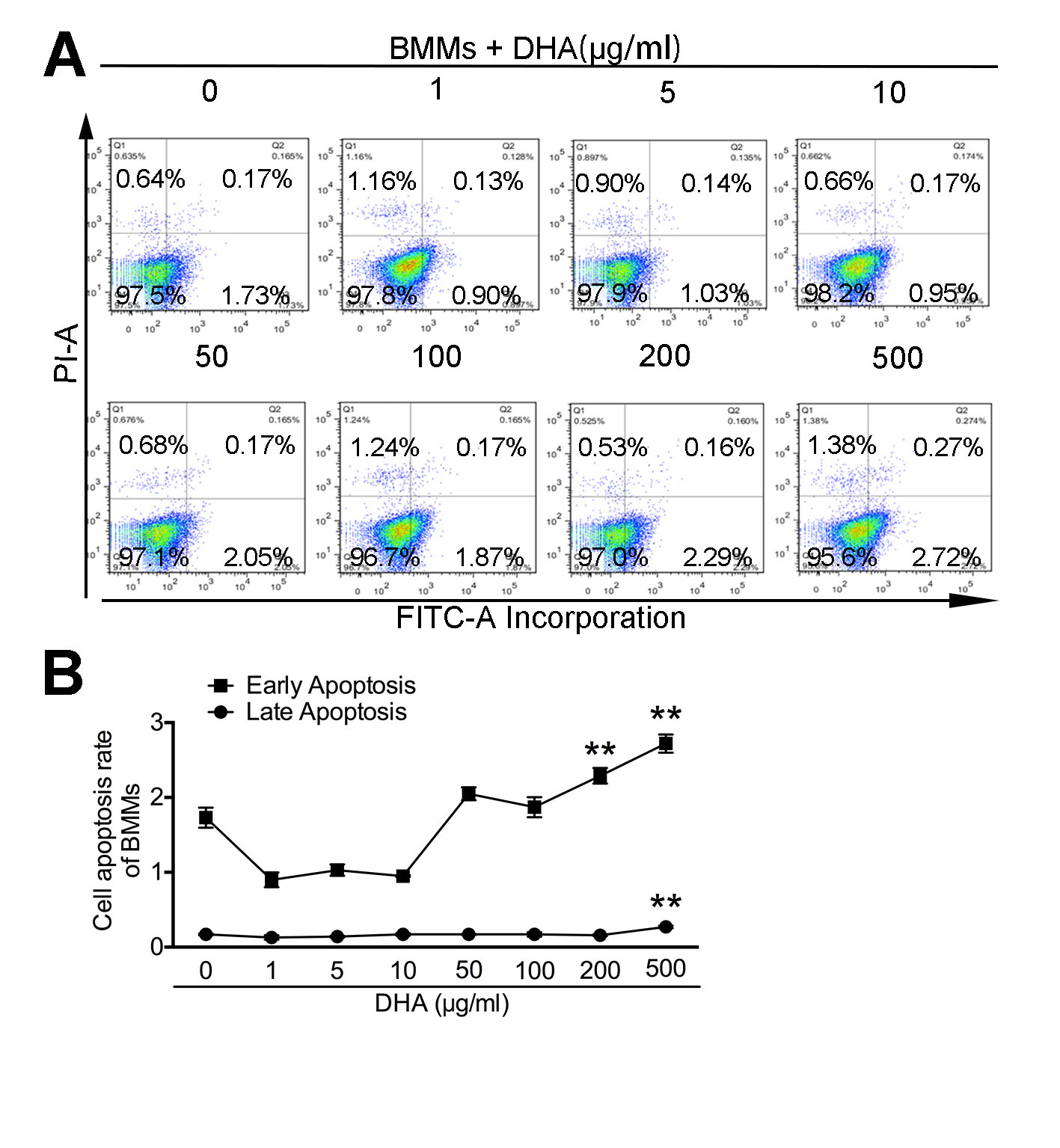


**Supplementary Fig. 6 Effects of DHA on cell apoptosis. (A)** FCM analysis of the apoptosis rate of BMMs treated with DHA for 72 h. **(B)** Quantitative analysis of the early and late stage apoptosis rates. The data in the figures represent the averages ± SD. Statistically significant differences between the treatment and control groups are indicated as * (*p*< 0.05) or ** (*p*< 0.01).
